# Comparing Early Eukaryotic Integration of Mitochondria and Chloroplasts in the Light of Internal ROS Challenges: Timing is of the Essence

**DOI:** 10.1128/mBio.00955-20

**Published:** 2020-05-19

**Authors:** Dave Speijer, Michael Hammond, Julius Lukeš

**Affiliations:** a Medical Biochemistry, AmsterdamUMC, University of Amsterdam, Amsterdam, The Netherlands; b Institute of Parasitology, Biology Centre, Czech Academy of Sciences, České Budějovice (Budweis), Czech Republic; c Faculty of Sciences, University of South Bohemia, České Budějovice (Budweis), Czech Republic; Duke University

**Keywords:** chloroplast, eukaryogenesis, mitochondria, reactive oxygen species

## Abstract

When trying to reconstruct the evolutionary trajectories during early eukaryogenesis, one is struck by clear differences in the developments of two organelles of endosymbiotic origin: the mitochondrion and the chloroplast. From a symbiogenic perspective, eukaryotic development can be interpreted as a process in which many of the defining eukaryotic characteristics arose as a result of mutual adaptions of both prokaryotes (an archaeon and a bacterium) involved. This implies that many steps during the bacterium-to-mitochondrion transition trajectory occurred in an intense period of dramatic and rapid changes. In contrast, the subsequent cyanobacterium-to-chloroplast development in a specific eukaryotic subgroup, leading to the photosynthetic lineages, occurred in a full-fledged eukaryote. The commonalities and differences in the two trajectories shed an interesting light on early, and ongoing, eukaryotic evolutionary driving forces, especially endogenous reactive oxygen species (ROS) formation. Differences between organellar ribosomes, changes to the electron transport chain (ETC) components, and mitochondrial codon reassignments in nonplant mitochondria can be understood when mitochondrial ROS formation, e.g., during high energy consumption in heterotrophs, is taken into account.

## Abstract (precis)

The early eukaryotic evolution was deeply influenced by the acquisition of two endosymbiotic organelles - the mitochondrion and the chloroplast. Here we discuss the possibly important role of reactive oxygen species in these processes.

## OBSERVATION

**FIG 1:**
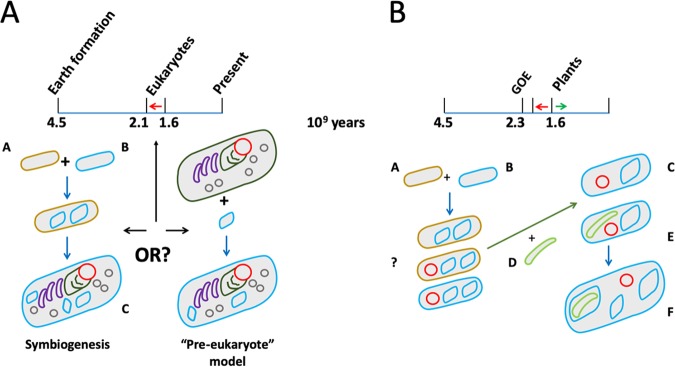
The evolution of eukaryotes, mitochondria, and chloroplasts. The timeline gives the relative timing of each. Of note, eukaryotes arrived “late,” i.e., after the great oxygenation event (GOE). (A) Symbiogenesis versus slow pre-eukaryote development with “late” bacterial acquisition. We defend symbiogenesis (see text). Timing of eukaryogenesis is uncertain, where it occurred sometime between 2.1 and 1.6 billion years ago (red arrow) (68). Mitochondria, blue; nucleus, red; endoplasmic reticulum membrane, dark green; Golgi apparatus, purple; peroxisomes, gray. (B) The origin of photosynthetic eukaryotes upon cyanobacterial uptake by a eukaryote cell. A, “Asgard archaeon”(brown); B, alphaproteobacterium (blue); C, eukaryote with nucleus (red) formed in response to endosymbiont arrival; D, cyanobacterium (green). Further symbiosis after development of LECA (green arrow): E, photosynthetic eukaryotes (plants and algae); F, example of secondary endosymbiosis, e.g., by uptake of red or green algae (resulting in a secondary plastid; green/blue), as found in, e.g., dinoflagellates, euglenids, and stramenopiles (67). The membranes of the eukaryote “turned blue” (“?” depicting complete replacement of the archaeal by bacterial membranes, as further explained in the text). All membranes are schematized as single membranes. Adapted and extended based on data from reference 9.

To explain the origin of eukaryotes and the role a premitochondrial endosymbiont played, we essentially have two contending scenarios: a gradual stepwise model and a symbiogenic model (1), though more mixed models have also been proposed. Basically, the first model has an amitochondriate eukaryote take up a bacterium related to present-day alphaproteobacteria, which was destined to become the mitochondrion. The second, the symbiogenic model, posits that the conversion of an archaeon and a bacterium to a eukaryote *was the result of their merger*; see Fig. 1A (2–10). Of note, these two models roughly correspond to the phagotrophic and syntrophic categories defined by O’Malley (11). We use “symbiogenic” instead, in order to stress the fact that many eukaryotic characteristics seem to have been the direct result of mutual adaptations. Recent, extensive overviews of practically all theories regarding eukaryotic origins and the role of the endosymbiont that was to become the mitochondrion (also referencing pioneering thinkers on endosymbiosis, like Mereschkowsky and Margulis) can be found elsewhere (12–14). The symbiogenesis concept, postulating that eukaryotic features can be explained by mutual adaptations of the partners involved, also implies that all eukaryotic characteristics originated *after* entry of the future endosymbiont. The concept emerged in the context of the hydrogen (6) and syntrophy hypotheses (15). Alternative symbiogenic theories, replacing the transfer of hydrogen from bacterium to archaeon by reversible transfer of energy-rich compounds between endosymbiont and host (resembling modern mitochondrial function) seem more likely in light of recent findings (10, 16).

Here, we would like to stress just two aspects: (i) that eukaryotes seem an even mixture of archaeal and bacterial characteristics (6, 9, 17, 18), and (ii) that original (archaeal) host membranes have been completely replaced by bacterial membranes (6). This membrane swap could, for instance, be explained by speculating that the eukaryotic inner membrane network evolved from bacterial outer membrane vesicle secretion inside a pre-last eukaryotic common ancestor (LECA) organism (19), which would also replace the outer membrane structures. Whatever the precise mechanisms, both aspects dovetail nicely with symbiogenic models. Symbiogenesis predicts different selective pressures during bacterium-to-mitochondrion conversion, compared with later cyanobacterium-to-chloroplast development. Here, we will use telltale evolutionary comparisons to see whether this is borne out. To perform such a comparison, we first need to reconstruct what role ROS formation might have played during the early stages of eukaryotic evolution.

### How internal ROS formation might have contributed to eukaryogenesis.

If full symbiogenesis is correct, or even if a more gradual process of eukaryogenesis occurred, we can assume that internal ROS formation by proto-mitochondria helped shape many eukaryotic characteristics *before* the uptake of a primordial photosynthetic cyanobacterium (the chloroplast-to-be), which gave rise to the plastid-bearing eukaryotes (20–22). Thus, the two bacteria encountered fundamentally different environments upon primordial entry. We postulate that the eukaryote that took up the cyanobacterium had evolved extensive capabilities of dealing with ROS challenges because of the prior mitochondrial establishment. What are universal eukaryotic characteristics that could (partially) be explained in the context of variable endogenous endosymbiotic ROS formation? They are listed in Table 1.

**TABLE 1:** Eukaryotic characteristics which might be linked to internal ROS formation

Eukaryotic adaptations possibly linked to mitochondrial ROS formation	Proposed rationale	References
Peroxisomes	ROS reduction in mitochondria	(24, 64, 69)
Enhanced antioxidant mechanisms/iron sequestration	ROS reduction	(62, 70, 71)
Meiotic sex^*a*^	ROS (intensifying Muller’s ratchet)	(34, 35, 37, 38)
Mitochondrial fusion-fission cycles	Mitochondrial repair	(72)
Mitochondrial genome reduction	Organellar DNA protection from ROS	(9, 53)
Mitochondrial transhydrogenase	Oxidative repair	(62, 73, 74)
Autophagy/mitophagy	Mitochondrial repair	(75)
Nuclear membranes/histones	ROS (nuclear DNA protection)	(9, 76)
Uncoupling proteins, carnitine shuttles	ROS reduction and protection	(77, 78)

a Eukaryotic sex has many possible uses, but here we discuss the possible rationale behind its *emergence*.

The LECA, apart from the “anti-endogenous ROS” adaptations listed in Table 1, also probably developed (somewhat) coordinated organelle/cell doubling, division, and separation, as well as specific organellar protein targeting machinery. It constituted a highly complex, metabolically versatile organism, i.e., oxidizing carbohydrates, amino acids, and fatty acids. Consequently, it could have accommodated the next endosymbiont more easily than the mitochondrial precursor (see below).

With regard to the protein targeting machineries, components of the endoplasmic reticulum (ER)-associated degradation (ERAD) transport systems are also used for mitochondria-associated degradation (MAD) and peroxisomal import (and possibly export) (23), evolutionarily linking ER, mitochondria, and peroxisomes (24, 25). During later acquisitions, this machinery was reused to develop the symbiont-derived ERAD-like machinery (SELMA) to get proteins into secondary (i.e., “algal”) plastids (25–27). The uptake of a cyanobacterium (the primordial chloroplast) led to a distinct import mechanism mostly composed of bacterial proteins (the TOC and TIC translocons), which guaranteed specificity in cells already containing other intracellular targets for protein delivery (28). Still, this mechanism resembles mitochondrial protein import in many aspects: it is ATP-dependent, occurs *after* translation, and uses different translocation complexes in each respective membrane, with specific membrane contact sites. In both cases, organellar proteins have amphipathic N-terminal signal sequences that are proteolytically removed upon entry. Whether these striking commonalities are because chloroplast import had to copy the existing eukaryotic machinery, or whether such a system is intrinsically superior, remains an open question.

Though the question of how the merger between an archaeon and a bacterium, giving rise to the mitochondrion, occurred is hotly debated, we do not discuss it here. Also, whether the uptake of the progenitor of chloroplasts was accidental or occurred using a (newly available) phagocytic process by a “Cryptista-like” cell (22) is not considered further. However, in light of the scenario depicted in Fig. 1B, we maintain that a careful comparison of evolutionary developments in mitochondria and chloroplasts should turn up many more traces of ROS adaptations and revolutionary changes in mitochondria than in chloroplasts. This is the focus of the remainder of our article. But before we embark on this comparison, some preliminary remarks with regard to organellar ROS generation are necessary. While mitochondria consume oxygen, chloroplasts produce it. Though oxygen itself can be reactive (abstracting electrons from organic compounds), it should not be seen as a kind of ROS, as its triplet ground state is relatively stable (29). However, highly reactive singlet oxygen (a bona fide example of a ROS) is abundantly produced by photosystem (PS) II of chloroplasts, but locally and efficiently quenched by β-carotene, tocopherol, or plastoquinone (30). In plants, chloroplasts produce more ROS than mitochondria, but this is due to the use of an alternative terminal oxidase in this organelle (31). The major species of ROS also seem to differ, with mostly hydrogen peroxide formed in chloroplasts and the more reactive superoxide (O_2_-.) and hydroxyl radicals (.OH) formed in mitochondria.

### Comparing ROS formation in latter-day mitochondria and chloroplasts.

Both mitochondria and chloroplasts are characterized by important and highly active electron transport chains (ETCs), with oxygenic photosynthesis relying on PS I and II in chloroplasts and the respiratory chain consuming molecular oxygen in mitochondria. Both are capable of substantial ROS formation. It is difficult to compare relative contributions, as measurements include different organisms, tissues, and kinds of ROS, as well as an abundance of diverse antioxidant mechanisms, as illustrated by the alternative plant oxidase mentioned above. Additionally, most reviews tend to be either plant-oriented (32) or animal-centered (33). Thus, we cannot deduce anything regarding relative oxidative pressure at the birth of these organelles from the fact that chloroplasts also exhibit extensive genome reduction (though less severe than found in mitochondria), which went hand in hand with large-scale gene migration to the nucleus. We speculate that the chloroplast’s precursor arrived in a cell already equipped with protein import machinery, a nuclear DNA-protective environment with superior, less error-prone DNA replication, and expression mechanisms that allowed better fine-tuning firmly in place. Last but just as important, the nuclear genome of eukaryotes seems to suppress mutational build up (Muller’s ratchet) by meiotic sex (34–38). In contrast, organellar genes remain relatively unprotected. So, even with relatively low ROS pressure, long-term cyanobacterial gene migration would be favored. As it happens, we have a model system for a much more recent arrival of a cyanobacterium as an endosymbiont in photosynthetic *Paulinella* species (22, 39). The plastid is often referred to as the “cyanelle” or chromatophore of these amoeboids, and indeed, its genome was reduced to about one-third of its original size compared to its modern free-living relatives (22). One can observe a consistent correlation between the *Paulinella* plastid genome, at nearly 1 Mb, which is at least 10-fold larger than a typical plastid genome, and the fact that it has existed as an endosymbiont for ∼8% of the time that chloroplasts have been around, as it was taken up 90 to 140 million years ago (40). These organisms indeed experience ROS stress under light conditions (41), yet we can only speculate whether this has functioned as an extra driving force in genome migration.

### Mitochondrial codon reassignment upon ROS stress.

In metazoan and some yeast lineages (which are closely related from a eukaryotic vantage point), ROS may have led to the antioxidant codon reassignment of Ile → Met (codon AUA) in the mitochondria (42). This sense-to-sense codon reassignment has occurred independently multiple times in these lineages (43), indicating it could have beneficial effects. It is not difficult to understand why, as it gives rise to a distinct accumulation of the easily oxidized amino acid methionine within inner mitochondrial membranes and in subunits of the complexes involved in oxidative phosphorylation (OXPHOS). The sulfur-containing amino acid methionine is normally oxidized to R- and S-methionine sulfoxide, which can be quickly reduced again by stereospecific methionine sulfoxide reductases (43). However, surprisingly, transgenic mice missing all reductases were *more* resistant to some forms of ROS stress (44). This again illustrates the difficulties of studying ROS adaptation in complex animals, in which early ROS challenges can induce effective antioxidant mechanisms, reflected in the concept of mitohormesis (45).

But why would this potentially beneficial feature be restricted to animals and yeasts? Metazoans are prime examples of high-energy-consuming heterotrophs and yeasts can have lifestyles leading to increased levels of ROS formation. Of note, mitochondrial codes in plants, along with their resident chloroplasts, do not display this antioxidant codon reassignment, which seems to indicate that local oxygen production as such does not determine ROS levels. Thus, this arrangement could be an example of high local ETC ROS pressure leaving evolutionary markers. Obviously, these codon reassignments developed after LECA formation and are not informative when it comes to the pressures encountered by the merged set of prokaryotes during the evolution toward LECA. However, important insights might be gained through comparison of mitochondria and chloroplasts regarding this transition, as well.

### Comparing ribosomes in mitochondria and chloroplasts.

Recently, van der Sluis et al. tried to reconstruct the evolution of mitoribosomes and the five mitochondrial OXPHOS complexes (46). Their impressive data set enables the dissection of mitochondrial evolution using structural and bioinformatic methods. Apart from the ongoing migration to the nucleus of bacterial genes encoding components of these molecular machines, both complexes of OXPHOS and mitoribosomes also have far more nuclear-encoded “extra” (supernumerary) subunits than their alphaproteobacterial ancestors, and these tend to be located on the *exterior* of the respective complexes. The authors then demonstrate that in both the mitoribosomal RNAs and hydrophobic core subunits of the OXPHOS complexes, destabilizing mutations are compensated by extra protein subunits. This process was completed in LECA for all universally conserved supernumerary proteins (∼75 novel subunits). This conclusion is borne out by the reconstructed mitoproteome (from a draft nuclear genome sequence) of the jakobid Andalucia godoyi (47). Jakobids, which have the largest mitochondrial genomes known so far (48), though displaying a few ancient (bacterial) mitochondrial features, thus cannot be seen as “transitional” (47).

This early phase, during which novel subunits were recruited, van der Sluis et al. call the constructive evolution stage. Next, and (mostly) restricted to metazoans, a reductive phase occurred, resulting in a gradual length reduction of the mitochondrion-encoded rRNAs and OXPHOS proteins, causing further intrinsic destabilization compensated by (extra) lineage-specific supernumerary proteins (46). That the reductions in rRNA gene length need to be compensated by additional proteins is nicely illustrated by the parasitic protist Trypanosoma brucei. Its mitoribosome has the smallest rRNAs, while containing most proteins, of all known ribosomes (49).

As discussed above, the authors argue for structural compensation of the mutationally destabilized mitochondrion-encoded components by the newly recruited, nuclear-encoded ribosomal and OXPHOS subunits. However, we can observe a gulf in this respect between chloroplasts and mitochondria. Despite seemingly low mutation rates, chloroplast genomes have likewise almost completely migrated to the nucleus but their ribosomes remained “bacterial” in structure, without destabilizing mutations and with only a few supernumerary subunits (50, 51).

Van der Sluis et al. hardly address the question of why this chasm exists, and neither do they speculate too much about the big difference observed for metazoan mitochondria (46). How can we explain the further reductive phase specifically observed in this group? The only hint of an explanation refers to “population genetic characteristics.” However, *both* chloroplasts and mitochondria form extremely small populations inside cells, which partly explains their accelerated genomic evolution rates. Both additionally possess membrane-bound redox complexes that likely form the basis for their genome retention, though the specific reasons why are debated (10, 52, 53). The nonexclusive retention theories stress the necessity for local translation because of extreme hydrophobicity and/or energetic centrality (see the “colocalization for redox regulation” hypothesis [52]) of the mitochondrion-encoded core OXPHOS subunits, with the corresponding genes having high GC content, relative to other genes in the same organism (53). Could this be related to the higher stability of GC bonds closing off DNA from environmental mutagens? Adenine depletion can indeed be observed during oxidative stress (54).

### The “Big Bang” of eukaryogenesis.

So, how to reconstruct the evolution of both organelles as well as the metazoan specificities in such a way that these divergent features start to make sense? Probably the best way to explain these different evolutionary paths is to take the “big bang” of eukaryogenesis seriously (6, 9, 10, 12, 14). As mentioned from the outset, we and many others posit that the eukaryotes started with the unlikely merger of an archaeon and a bacterium in an instance of symbiogenesis (8, 9, 12, 16, 55, 56). Symbiogenic models assume a rapid adaptive period (9). The uptake of nascent mitochondria allowed “expensive” eukaryotic inventions to be payed for by the efficient ATP generation using their respiratory chains on extended *internal* membranes (57). We posit that efficient ATP production, using *alternating* substrates (24), is accompanied by strongly enhanced toxic ROS formation on the inside of the cell, close to the membrane-linked (pre-) mitochondrial genome (58). Why “enhanced ROS formation”? Because the oxidation of carbohydrates, proteins, and lipids differs in the relative involvement of complex I (NADH dehydrogenase), such that the versatile nature of the new cell might lead to more ROS formation by the ETC (24). This highly unstable temporary state would create selective forces that could explain the large-scale migration of endosymbiont genes to a nuclear “safe-haven,” evolving under these selective forces, as well as numerous destabilizing mutations in the remaining mitochondrion-encoded ribosomal RNAs and OXPHOS subunits. This unstable environment characterizes important aspects of the road to LECA. Likewise, the further mitochondrial genome reduction with supernumerary compensation in some protists, metazoans, and yeasts can possibly be explained by high energy consumption, leading to increased ROS formation, in heterotrophs. This already indicates a first crucial difference with the uptake of the future chloroplast.

In LECA, the “host” cell is completely changed from the original archaeon, which also helps explain the sharply contrasting outcome in the case of subsequent chloroplast uptake. Many antioxidant mechanisms are now available, while fine-tuning of metabolism (due to a higher level of regulation and feedback control) in response to ROS production can be implemented (Table 1). This diverging evolutionary path should also be seen in light of the fact that *endogenous substrate and ATP production* by the new endosymbiont (plants becoming autotrophs) makes the highly efficient ATP generation in mitochondria, characteristic of many heterotrophs, less crucial. That plants are not limited at the energy level in the way of metazoans is nicely illustrated by calculations for the illuminated leaves of C3 plants. Here, mitochondrial ATP synthase is predicted to contribute less than 1/5 of total ATP, the rest coming from chloroplast thylakoid ATP synthase (59). Accordingly, van der Sluis et al. note that mutation rates in the mitochondrial genome of plants are exceptionally low, potentially as a result of lower ROS formation from reduced ATP demand (46).

Thus, the synergistic combination of the two driving forces of highly efficient mitochondrial ATP generation plus initially enhanced endogenous ROS formation gave rise to the accelerated evolution of eukaryotes. This explains the constructive evolution stage. Such accelerated evolution might also have contributed to the fact that mitochondrial proteomes are largely encoded by genes without identifiable archaeal or alphaproteobacterial provenance (18, 60), although ∼2 billion years of independent evolution and extensive bacterial horizontal gene transfer must also be taken into account. LECA’s mitochondrial catabolic contribution (highly efficient ATP generation resulting in high ROS formation) probably remained the major evolutionary influence in certain heterotrophic modes, i.e., those with limiting food resources, explaining the reductive phase (46) in organisms dependent on such modes. This led to some highly efficient unicellular and multicellular lineages, with streamlined, catabolically adaptable mitochondria, which optimize the ATP/ROS generation trade-off. A nice example can be found in animals, where the peroxisomes only perform β-oxidation on very long-chain fatty acids, while all other fatty acids are oxidized in a more efficient way in the mitochondria (61), thereby allowing the most ATP at the lowest “ROS costs” (62). Alternatively, yeast has moved all fatty acid oxidation to peroxisomes, illustrating different trade-offs in different eukaryotic lineages (63, 64). Peroxisomes are most easily understood as eukaryotic inventions to lower mitochondrial ROS formation (24), and recent findings strongly suggest that they developed specifically in response to the entry and incorporation of the premitochondrion (65, 66).

In conclusion, timing of the encounter, and the nature of the two organisms involved, is reflected in many aspects of present-day cellular architecture. The original merger between equals seems highly unlikely to succeed. However, when it succeeded, it turned out to be incredibly fruitful, as eukaryotes attest. All subsequent fascinating (real eukaryotic) acquisitions (67), even that of a cyanobacterium ending up as a chloroplast, needed far fewer adjustments and have occurred much more often.
